# Adherence to triage among women with HPV-positive self-collection: a study in a middle-low income population in Argentina

**DOI:** 10.3332/ecancer.2020.1138

**Published:** 2020-11-10

**Authors:** Melisa Paolino, Juan Gago, Anabella Le Pera, Oscar Cinto, Laura Thouyaret, Silvina Arrossi

**Affiliations:** 1 Centro de Estudios de Estado y Sociedad/Consejo Nacional de Investigaciones Científicas y Técnicas, Sánchez de Bustamante 27, Buenos Aires 1193, Argentina; 2 Department of Population Health, School of Medicine, New York University (NYU), 550 1st Avenue, New York, NY 10016, USA; 3 Centro de Estudios de Estado y Sociedad, Sánchez de Bustamante 27, Buenos Aires 1193, Argentina; 4 Ministerio de Salud Pública de Tucumán, Av. República del Líbano 956, San Miguel de Tucumán, Tucumán, Argentina; 5 Programa Nacional de Prevención de Cáncer Cervicouterino /Instituto Nacional del Cáncer (Argentina), Julio A, Roca 781, Piso 9, Buenos Aires 1067, Argentina; 6 Centro de Estudios de Estado y Sociedad/Consejo Nacional de Investigaciones Científicas y Técnicas, Sánchez de Bustamante 27, Buenos Aires 1193, Argentina; * Retired; a https://orcid.org/0000-0002-8649-1570; b https://orcid.org/0000-0002-5071-0938

**Keywords:** cervical cancer, human papillomavirus DNA test, self-sampling, lost to follow up, Argentina

## Abstract

**Introduction:**

Screening for cervical cancer (CC) prevention has substantially changed with the introduction of human papillomavirus (HPV) tests. This technology compared to cytology has increased the detection of pre-malignant and malignant cervical lesions in real-world programmes in different settings. Very importantly, through self-collection, HPV testing can reduce barriers to screening and increase coverage. However, when using HPV self-collection, triage tests are a key step in the CC prevention process, and high adherence to triage has been difficult to obtain in low-middle income settings. The aim of this study was to measure adherence to triage among women with HPV+ self-collection and analysed factors associated with this adherence in a middle-low resource setting in Argentina. We also evaluated key indicators related to the implementation of the HPV self-collection strategy.

**Methods:**

We analysed data on screening/triage/diagnosis/treatment from women aged 30+ who performed self-collection between 2015 and 2017 (*n* = 15,763), in the public health system in Tucuman, Argentina. We analysed secondary data from the national screening information system. The primary outcomes were: 1) adherence to cytology triage within the recommended timeframe (120 days) and 2) overall adherence to cytology triage including data at 18 months after screening. Multivariable regression was used to examine the association between age group, year of the screening test, record of the previous Pap-based screening and health insurance status with adherence to triage test as a primary outcome. We reported odds ratios, 95% confidence intervals and *p*-value of 0.05, which was considered the threshold for *p*-values).

**Results:**

We analysed data of 2,389 HPV+ women. The overall adherence to triage at 18 months was 42.9%. The percentage of women completing cytology triage within the recommended timeframe of 120 days was lower (25.2%). Women with the record of a previous Pap-based screening had 1.86 times the odds of having a triage compared to women without a record of a previous Pap-based screening (95% CI: 1.64–2.64, *p* <0.001). Furthermore, the probability of having triage at the recommended timeframe was higher among women who were older and women with public health insurance.

**Conclusions:**

Our results showed that adherence to triage in the recommended timeframe was low. In addition, the probability of having triage at the recommended timeframe was higher among women with a record of a previous Pap-based screening, a proxy of the use of health services. Our results showed that adherence to triage in the context of the HPV-self-collection strategy is challenging. The implementation of alternative approaches that might facilitate adherence to triage should be further investigated.

## Introduction

Cervical cancer (CC) can be prevented using currently available technology, which has led WHO to make a global call for action towards the elimination of CC [1]. Even though it is almost entirely preventable, it is the second leading cause of cancer death among women in low and middle income settings [2]. High mortality is related to problems in the continuity of the prevention process, including low screening coverage and loss to follow-up, and treatment [3–5].

Screening for CC prevention has substantially changed with the introduction of human papillomavirus (HPV) tests. This technology compared to cytology has increased the detection of pre-malignant and malignant cervical lesions in real-world programmes in different settings [6–8], and it has been proven effective to reduce CC incidence and mortality [9]. Very importantly, through self-collection, HPV testing can reduce barriers to screening and increase coverage [10–12], especially among hard-to-reach women who are at higher risk of CC [13]. The method is highly accurate [12], acceptable for women in different countries [10–14] and effective to increase screening uptake [11, 12, 15].

However, HPV testing is only effective if the corresponding follow-up and treatment are provided to all women [16]. HPV-positive (HPV+) tests only inform about infection with oncogenic HPV, so triage tests are needed to identify those women who need additional diagnostic procedures. Many countries use cytology for triage, including Argentina, and it is one of the recommended methods by WHO guidelines [17]. In Argentina, during a screening visit at a health centre, providers perform both HPV and cytology tests, but cytology is only read if the HPV test is positive [18]. However, it is not possible to take both the HPV test and cytology when women self-collect samples at home, and women with HPV+ self-collection are referred for triage at the health centre, creating an additional step in the screening process.

Studies assessing adherence to triage in programmatic contexts in low and middle resource settings are scarce, but few available studies have shown that adherence to triage for women with self-collected tests is a challenge [8, 13, 19–21]. A study in Nicaragua showed that the odds of completing triage were almost three times higher for women who had a provider-collected sample compared to women who collected their own sample for HPV testing [20]. In Jujuy, Argentina, only 18% of women with self-collected tests completed triage 60 days after an HPV+ result [21]. This loss to follow-up is a reflection of structural, subjective and health system determinants of access to healthcare services. For example, previous studies have shown that long delays in delivering test results, long waiting times or limited appointments availability have a negative impact on the completion of follow-up [16, 21–23]. The loss to follow-up and its determinants –historically faced by cytology-based screening programs – needs to be evaluated when implementing an HPV-based screening program. Otherwise, it will not be possible to achieve high levels of adherence to follow-up and treatment, and as a result, the effectiveness of the HPV test to prevent CC will be reduced.

Therefore, there is a need to provide evidence about adherence to triage among women with HPV self-collected tests in different settings in order to devise strategies aimed at increasing the completion of follow-up. In Argentina, HPV-testing was introduced in 2012 through the Jujuy Demonstration Project [8]. In 2015, HPV-testing was scaled-up to four additional provinces, including the province of Tucumán, located in Northwest, which is one of the poorest regions in Argentina. The provincial public health system is comprised of a network of public hospitals and primary healthcare (PHC) units that include 7 third-level hospitals, 24 second-level hospitals and more than 300 PHC units [24]. For the uninsured, health services are provided free of cost, including screening, diagnosis and treatment. In Tucuman, programmatic, population-based self-collection was implemented in 2015. HPV test (Hybrid Capture 2; Qiagen, Germantown, MD, USA) was introduced as primary screening for women aged 30 years and older attending the public health system.

Our study analysed data about HPV self-collection in Tucuman from 2015 to 2017. We measured adherence to triage among women with HPV+ self-collection and analysed factors associated with this adherence. We also evaluated key indicators related to the implementation of the HPV self-collection strategy.

## Methods

### HPV-based screening procedures

In Tucumán, HPV self-collection is offered by community health workers (CHWs) during home visits or community-health meetings. All HPV samples are self-collected following instructions given by the CHWs. HPV+ women have to complete a triage test (cytology) at a health centre. Women who are HPV+ and have an abnormal cytology result (atypical squamous cells of undetermined significance – ASC-US – atypical squamous cells cannot exclude high-grade lesions – ASC-H – low-grade lesions, high-grade lesions or cancer) are referred for colposcopy/biopsy and treatment if needed. HPV-negative (HPV) women are recommended re-screening in 5 years. HPV+/normal cytology women are recommended re-screening in 18 months [18]. Women younger than 30 continue to be Pap-screened.

### Data source

Since 2015 in Tucumán any instance of screening, diagnosis, or treatment using public health services has been registered in the national screening information system (SITAM, by its initials in Spanish) [25]. SITAM is a unified database that collects data about screening, diagnosis and treatment of all women attending the public health system. The HPV laboratory used SITAM to manage samples at entry; the samples of individuals that did not comply with the recommended age range or screening frequency were not processed.

We extracted data from SITAM for the purpose of this analysis. We analysed the SITAM database containing records of all women aged 30 years and older screened in Tucuman using HPV self-collection from 2015 to 2017 (*n* = 15.757) and data until June 2019 for follow-up (triage, diagnosis and treatment). Colposcopies, biopsies and treatments not registered in SITAM were considered lost to follow-up, including those carried out in private services without confirmation of that information by the provincial program. The data are accessed by authorised healthcare workers and researchers. A non-disclosure agreement of the personal data is signed before a user and password is provided to access to the databases.

### Independent variables and outcomes

Independent variables considered for this study were: age (30–34, 35–44, 45–54, 55–64,65+); year of screening test (2015, 2016 and 2017); record of previous Pap-based screening (Yes/No) and health insurance status (public, private).

The primary outcomes were: 1) adherence to cytology triage within recommended timeframe – calculated as the percentage of HPV+ women with cytology triage at 120 days after screening and 2) overall adherence to cytology triage including data at 18 months after screening. Both outcomes were re-corded as dichotomous variables.

In addition, we evaluated the following key programs indicators:

- Positivity: Number of HPV+ women/total women with self-collected tests.
- Percentage of HPV+ women with abnormal cytology (ASCUS+): Number of HPV+ women with abnormal cytology (ASCUS+)/total HPV+ women with cytology triage test.
- Adherence to colposcopy: Number of women with colposcopy/total women referred to colposcopy.
- Adherence to treatment: Number of women with the histological confirmed high-grade lesion (CIN2+) with treatment/total women with CIN2+.

Detection rate (DR) per screening women: Number of women with the histological confirmed high-grade lesion (CIN2+)/ total women screened with self-collection.

DR per colposcopies: Number of women with the histological confirmed high-grade lesion (CIN2+)/total women with colposcopies.

### Statistical analysis

We performed a set of descriptive statistics and logistic regression models.

We performed a descriptive analysis of the demographic characteristics of the women who performed self-collection during 2015–2017.

Also, a multivariable regression was used to examine the association between age group, year of screening test, record of previous Pap-based screening and health insurance status (public or private) with adherence to triage test as a primary outcome. From this, we reported odds ratios with 95% confidence intervals and *p*-value which was used to determine the probability under this statistical model that the differences between the groups would be equal to or more extreme than its observed value, assuming that null is true. R-Statistical software and R-Studio were used to perform the analysis.

### Ethical statement

This protocol has been approved by the Tucuman Institutional Review Board (Protocol number Expt 583/623/D/2018). The identity of participants has been preserved by de-identification of the databases. Verbal informed consent has been obtained according to the national regulations for standard medical practices (Patient’s Rights Act 26.529). The requirement of specific consent does not apply for statistical analysis of aggregated de-identified data (Res. 1480/2011 Ministry of Health).

## Results

Between January 2015 and December 2017, 15,763 women aged 30 and older were screened with HPV self-collection (40.3% of total HPV-testing; *n* = 15,763/39,109). About half of the women screened in these 3 years were tested in 2015 (*n* = 7,915/15,763). A total of 2,389 women were HPV positive (15.2%). Among HPV+ women the mean age was 41.7 (SD 9.2), only 15.4% had a record of a previous Pap-based screening and 76.7% had public health insurance (Table 1).

**Figure 1.**
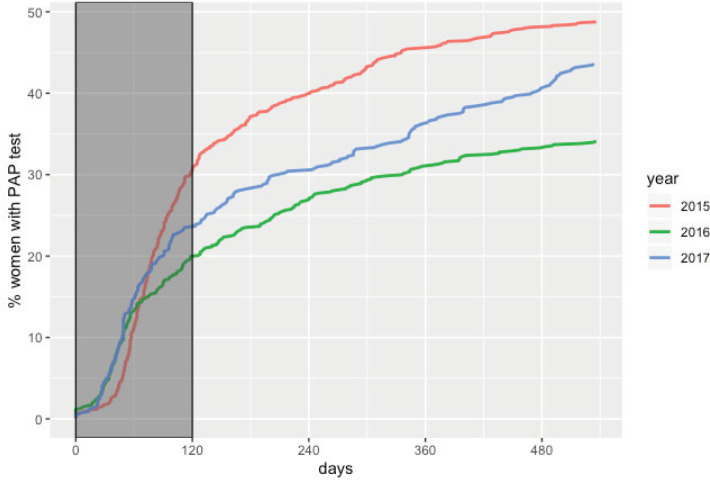
Adherence to triage by year of screening. Tucumán 2015–2017.

**Figure 2.**
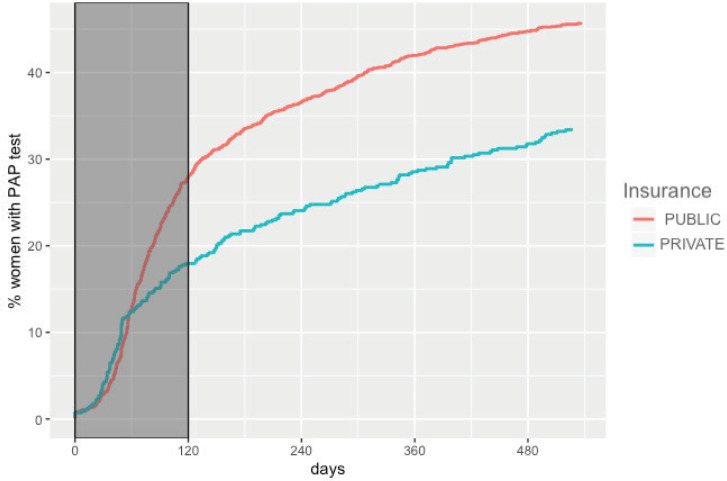
Adherence to triage by health insurance status. Tucumán 2015–2017.

**Figure 3.**
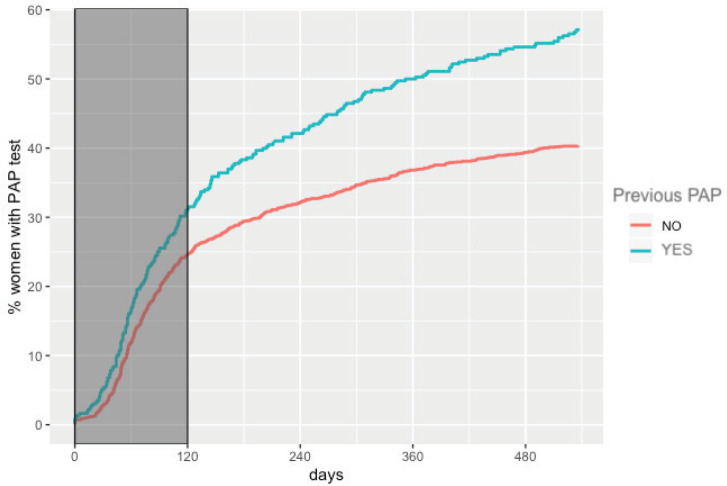
Adherence to triage by previous Pap-based screening. Tucumán 2015–2017.

The overall adherence to triage among HPV+ women screened between 2015 and 2017 was 42.9% (*n* = 1,026). This figure was different for each year: 48.8%, 34.1% and 43.5% for women screened in 2015, 2016 and 2017, respectively (Figure 1). Overall adherence to triage was the highest among women with public health insurance (45.8%, Figure 2) and among women with previous Pap-based screening (57.3%, Figure 3). Adherence to triage within recommended timeframe (120 days) was 25.2%. This percentage was 30.8% for those tested in 2015, 20.2% for women tested in 2016 and 23.5% for 2017 (Figure 1). Around 28.2% of women with public health insurance adhered to triage within 120 days versus 18.0% among women with private health insurance (Figure 2) and 31.3% of women with a previous Pap-based screening adhered to triage within 120 days versus 24.8% without previous Pap-based screening (Figure 3).

Among those women with positive triage in 2015–2017 (*n* = 365), 60% (*n* = 219) proceeded to perform a colposcopy, of which 72.2% (*n* = 158/219) resulted in pathological results (Table 2). A total of 92.4% of these pathological colposcopies had a record of a biopsy. The number of CIN2+ cases confirmed by histological results in the period studied was 78, resulting in a CIN2+DR of 4.95 per 1,000 screened women (*n* = 78/15,763) and 356 per 1,000 colposcopies (*n* = 78/219). About 91% of women with CIN2+ registered treatment (Table 2).

The results of the multivariate regression testing the association between adherence to triage at recommended timeframe and women characteristics are shown in Table 3. Women HPV-tested during 2016 were less likely to have triage when compared to those screened during 2015 (OR: 0.49; 95% CI: 0.42–0.64, *p* < 0.001). Women with a record of a previous Pap-based screening had 1.86 times the odds of having a triage compared to women without a record of a previous Pap-based screening (95% CI: 1.64–2.64, *p* < 0.001). Furthermore, the probability of having triage at the recommended timeframe was higher among older women and women with public health insurance. Multivariate regression testing the association between overall adherence to triage and women characteristics reported similar results (Table 4).

## Discussion

Although analysis of adherence to follow-up in CC screening has been widely investigated, there is limited evidence about adherence to triage in low-middle income settings where HPV self-collection is used for primary screening. Our study provides data that is key for countries considering the incorporation of HPV self-collection into their CC prevention programs.

In our study, the overall adherence to triage at 18 months was 43%. The percentage of women completing cytology triage within the recommended timeframe of 120 days was lower (around 25%). A higher percentage of adherence to triage has been reported in clinical trials and in organised CC screening programmes in high-income settings [12, 26, 28]. In 20 trials, adherence to follow-up among women with self-samples that tested positive for HPV was reported, and on average, 80.6% (41%–95%) had a follow-up examination [12]. In addition, in organised programs in the Netherlands [27] and Denmark [28] the reported percentage of triage was 77% and 90%, respectively. The highest adherence rates were found in studies with direct referral to colposcopy, and/or intensive follow-up protocols (e.g., in Denmark HPV test results and follow-up recommendations were mailed to the women and their GPs). However, limited compliance to cytology triage has been reported among women with self-collected test from Argentina [13, 21], France [19], Italy [29] and Nicaragua [20]. In a study carried out in France among non-attenders of low socio-economic level, 41% of women had a Pap smear after a positive HPV self-collected test [19]. A study in Nicaragua carried out to analyse the key characteristics of an HPV-screening programme showed that 54% of women adhered to triage [20]. In Argentina, a study showed that 18% and 30% completed triage 60 and 120 days after a positive HPV result, respectively [21]. Studies that analysed why screen-positive women failed to complete follow-up and treatment in context of cytology-based programmes found that in most cases this was due to problems related to the health services organisation [22, 23, 30] (i.e., delays or failures in result delivery, lack of appropriate guidance concerning the steps to follow after receiving a positive test, problems with appointments, and long waiting times). Several studies suggest that communication of cervical cytology results to women may be delayed, not conveyed, or misunderstood, which may lead to loss of recommended follow-up [22, 23, 31]. In addition, subjective reasons (e.g., fear or denial regarding the disease) and social factors, including work and domestic organisation and problems with transportation, were also reported [21–23, 32]. Similar problems could explain non-adherence to triage in the new context of screening with self-collection. This indicates that there is a critical need for comprehensive interventions to improve the delivery of results and to organised services according to women’s needs.

In our study, only 15% of HPV+ women who performed self-collected tests had a history of previous screening. In addition, women with previous screening were almost two times more likely to adhere to triage. Record of previous screening has been used as a proxy of the use of health services [33, 34]. In general, women who have increased contact with the healthcare system are more likely to adhere to screening/follow-up recommendations [35, 36]. For example, Luque *et al* [36] found that the number of previous medical office visits was a factor associated with adherence to CC screening in the USA. A past doctor’s visit was also a significant determinant of the probability of having a recent Pap smear in Latin America [37, 38]. A study carried out in five Latin American Countries showed that compared to women who had not had a recent doctor’s visit, the probability of being Pap screened was higher among those who had a medical visit: 48% higher in Bolivia, 241% higher in Brazil, 98% higher in the Dominican Republic; 77% higher in Guatemala and 94% higher in Nicaragua [37]. Thus, our results suggest that although HPV self-collection is reaching women with low access to screening services, adding a visit for triage constitutes a problem, as known barriers to screening [38–41] (i.e., lack of time to go to the health centre, problems with booking appointments, embarrassment or perceived pain of the Pap procedure)^,^ continue to act as negative determinants of the follow-up process. Thus, in a self-collection modality, factors that limit access to healthcare services will need to be addressed.

Several studies [42–47] are evaluating triage alternatives (e.g., Methylation, HPV genotyping, etc.) for the management of women with high-risk HPV infections that will reduce the number of clinic visits and reduce the number of steps in the diagnostic confirmation process [43]. In the meantime, it is necessary to devise strategies that facilitate women's access to triage. If self-collection is offered at home, the delivery of results and referring patients for follow-up is challenging, as the ‘point of entry’ is not at a health centre. The use of mHealth technologies to send reminders to women and health providers could be a viable tool to reduce the time from screening and triage and improve patient-provider communication. In Jujuy, during 2019 an effectiveness-implementation hybrid type-I trial was carried out to evaluate the effectiveness of a multi-component mHealth intervention to increase adherence to triage among women with HPV+ self-collected tests (ATICA project) [48]. Strategies that reduce the number of visits should be also considered. For example, ‘colposcopy and treat’ approach, combining colposcopy and treatment with thermal ablation of the cervical abnormalities in a single visit, provides a promising algorithm to improve patients’ compliance [49–51]. Implementation strategies of this alternative approach should be further investigated. 

Our study showed that adherence to colposcopy was higher than adherence to triage (60% versus 43%). In addition, more than 90% of women with CIN2+ had treatment. Similar levels of adherence to diagnosis and treatment have been reported in Jujuy (70% and 81%, respectively) [8]. This level of adherence in Jujuy was assured by implementing a navigator program to provide support for women and reduce the barriers to follow-up and organising a diagnosis and treatment network [52]. In Tucumán similar strategies have been implemented with women HPV + /cytology ASCUS + since 2015. However, a navigation programme to all HPV+ women as a public health strategy is difficult to sustain as a high proportion of all screened women would need to be contacted (around 15%). As mentioned above, loss to follow-up mostly took place in the triage stage, which is to say the first step after screening. This result is consistent with results reported by other studies in which the highest level of drop-out occurred before the diagnosis stage [22]. Thus, the low level of adherence to triage could be considered an indicator of flaws in the transferal of information and responsibilities between the patient and providers, among providers, and across organisational settings [53]. Conversely, the highest levels of adherence to colposcopy and treatment might be the result of a heightened sense of urgency by providers to refer women with abnormal cytology or/and CIN2+ diagnosis and to alert women of that urgency, as well as a good level of organisation of diagnosis and treatment networks to attend these cases. The identification of professionals responsible for the follow-up of women with HPV+ self-collection, the implementation of systems of notifications/alarms for these cases and the reformulation of the referral and counter-referral system (adapted to the new context of CC screening with HPV self-collection) are possible strategies to improve the trajectory of women across the continuum of care.

In Tucuman, the overall CIN2+ DR was around 5 per thousand screened women, lower than the rate reported in the province of Jujuy during programmatic scaling up of HPV self-collection (7/1,000) [8]. Similar CIN2+ DR among women with self-collection was also reported in Italy [29] and Greece [54]. In Tucumán, CIN2+ DR among women who did perform colposcopies was higher than the rate observed in Jujuy (356/1,000 versus 333/1,000, respectively) [8] and other studies [29, 55]. For example, Louvanto *et al* [55], in Canada, reported a CIN2+ DR of 340/1000 colposcopies [55]. Therefore, the reduction observed in CIN2+ DR during the scaling up of self-collection in Tucuman might probably not due to the accuracy of self-collection but due to the loss of follow-up.

In contrast to what has been found by previous studies [21, 23, 56], women with private/social health insurance were less likely to adhere to follow-up than women with public health insurance. However, these data should be interpreted with caution, as under-reporting of diagnosis procedures and treatments could partially explain this result. In effect, diagnosis and treatment performed by providers from the private health sector and the social security system are not routinely registered in the screening information system (SITAM). This highlights the importance of having a comprehensive information system that includes information about screening/diagnosis/treatment from all health sectors. This not only will reduce misinformation, but will also improve analysis of key programme indicators, and allow more efficient use of resources.

### Study limitations

Notwithstanding the useful information provided by this study, a number of limitations exist. First, the variables included in the analysis were those that the National Cervical Cancer Prevention Programme collects as routine data. Therefore, other variables not registered in the information system might be influencing the triage process. Second, this study is observational, and it is not conclusive evidence of the causal relationship between the variables analysed and the outcome. Furthermore, the level of adherence to triage might be under-estimated, as SITAM does not register diagnosis and treatment procedures of women who opted to continue their follow-up with private health providers or providers from the social security system.

## Conclusion

Our results showed that adherence to triage in the recommended timeframe was low. However, adherence to colposcopy and treatment was higher than adherence to triage. In addition, the probability of having triage at the recommended timeframe was higher among women with a record of a previous Pap-based screening, a proxy of the use of health services. Our results showed that adherence to triage in the context of the HPV-self-collection strategy is challenging, especially among women who are screening under-users. Given the impact that adequate follow-up and treatment have on the reduction of CC mortality, health system coordination is needed to guarantee adequate referral of HPV+ women. The implementation of alternative approaches that might facilitate adherence to triage should be further investigated.

## List of abbreviations

ASC-US: atypical squamous cells of undetermined significance
CC: cervical cancer
CHWs: community health workers
CIN2: cervical intraepithelial neoplasia grade 2
DR: detection rate
HPV: human papillomavirus
Pap: Papanicolaou
PHC: primary healthcare
SITAM: National Screening Information System (SITAM, by its initials in Spanish)
WHO: World Health Organization

## Funding declaration

No funding was received for this work.

## Conflicts of interest

The authors declare that they have no conflict of interest.

## Figures and Tables

**Table 1.** Socio-demographic characteristics of HPV positive women. Tucumán 2015–2017.

	Total HPV+ women		HPV positive women with triage		HPV positive women without triage	
	*n*	%	*n*	%	*n*	%
Total	2,389	100.0	1026	100.0	1,363	100.0
**Age**						
Mean (SD)	41.7 (9.2)		41.6 (9.3)		41.7 (9.1)	
30–34	624	26.1	269	26.2	355	26.0
35–44	993	41.6	424	41.3	569	41.7
45–54	475	19.9	200	19.5	275	20.2
55–64	267	11.2	113	11.0	154	11.3
65+	30	1.3	20	1.9	10	0.7
**Health insurance**						
Public	1832	76.7	839	81.8	993	72.9
Private	557	23.3	187	18.2	370	27.1
**Previous Pap**						
No	2021	84.6	815	79.4	1206	88.5
Yes	368	15.4	211	20.6	157	11.5
**Year of screening**						
2015	1083	45.3	529	51.6	554	40.6
2016	757	31.7	258	25.1	499	36.6
2017	549	23.0	239	23.3	310	22.7

**Table 2.** Screening performance indicators.

	2015–2017
Total women 30+ with self-collection	15,763
Total HPV+ women	2,389
Positivity (%)	15.2
**Follow up**	
Adherence to triage: HPV+ Women with triage^a^ (%)	44.4
Adherence to cytology triage: HPV+ women with cytology triage (%)	42.9
HPV+ Women with abnormal triage test (*n*)	365
Positive women with colposcopy (*n*)	219
Adherence to colposcopy: Positive women with colposcopy (%)	60.0
Adherence to treatment: Women with CIN2+ with registered treatment (%)	91.0
**Detection rate by screening**	
CIN 2+^b^	77
*CIN 2*	*17*
*CIN 3*	*24*
*CA*	*36*
**CIN2+detection rate (per 1000 screened women)**	4.95
**CIN2+detection rate (per 1000 colposcopies)**	356.2

a Include 34 women that were referred directly to colposcopy

b Include women with histological confirmed CIN 2, CIN 3 and Carcinoma

**Table 3.** Multivariate logistic regression. Variables associated with having cytology triage at 120 days. Tucumán Province, Argentina.

	OR	95%CI	*p*-value
**Age**			
30–34	1 (ref.)	-	
35–44	0.97	(0.79–1.20)	0.810
45–54	0.86	(0.71–1.19)	0.340
55–64	0.81	(0.68–1.26)	0.265
65+	3.05	(1.46–7.35)	0.015^a^
**Year of screening**			
2015	1 (ref.)		
2016	0.49	(0.42–0.64)	<0.001^a^
2017	0.78	(0.68–1.12)	0.099
**Previous Pap**			
No	1 (ref.)		
yes	1.84	(1.64–2.64)	<0.001^a^
**Health insurance**			
Public	1 (ref.)		
Private	0.60	(0.50–0.82)	<0.001^a^
* Constant est.*	0.037		0.001^a^

Standard errors: MLE

a *p*-value statistically significant

**Table 4.** Multivariate logistic regression. Variables associated with having cytology triage at 540 days. Tucumán Province, Argentina.

	OR	95%CI	*p*-value
**Age**			
30–34	1 (ref.)	-	
35–44	0.98	(0.79–1.21)	0.832
45–54	0.93	(0.72–1.19)	0.553
55–64	0.93	(0.69–1.26)	0.659
65+	3.19	(1.47–7.35)	0.043^a^
**Year of screening**			
2015	1 (ref.)		
2016	0.53	(0.42–0.65)	<0.001^a^
2017	0.88	(0.68–1.12)	0.031^a^
**Previous Pap**			
No	1 (ref.)		
yes	2.09	(1.65–2.64)	<0.001^a^
**Health insurance**			
Public	1 (ref.)		
Private	0.64	(0.50–0.82)	<0.001^a^
*Constant est.*	0.037		0.001^a^

Standard errors: MLE

a *p*-value statistically significant
